# Supplementary material for: Comprehensive Annotation and Functional Exploration of MicroRNAs in Lettuce
Source: Front Plant Sci. 2021 Dec 24;12:781836. doi: 10.3389/fpls.2021.781836 (PMC8739914; doi:10.3389/fpls.2021.781836)
Supplement: Supplementary file 3 [file Data_Sheet_9.ZIP › GSM2230752.plot/Lsa-miRN518_Lsat_1_v5_gn_8_2701.1_771_TPlot.pdf]

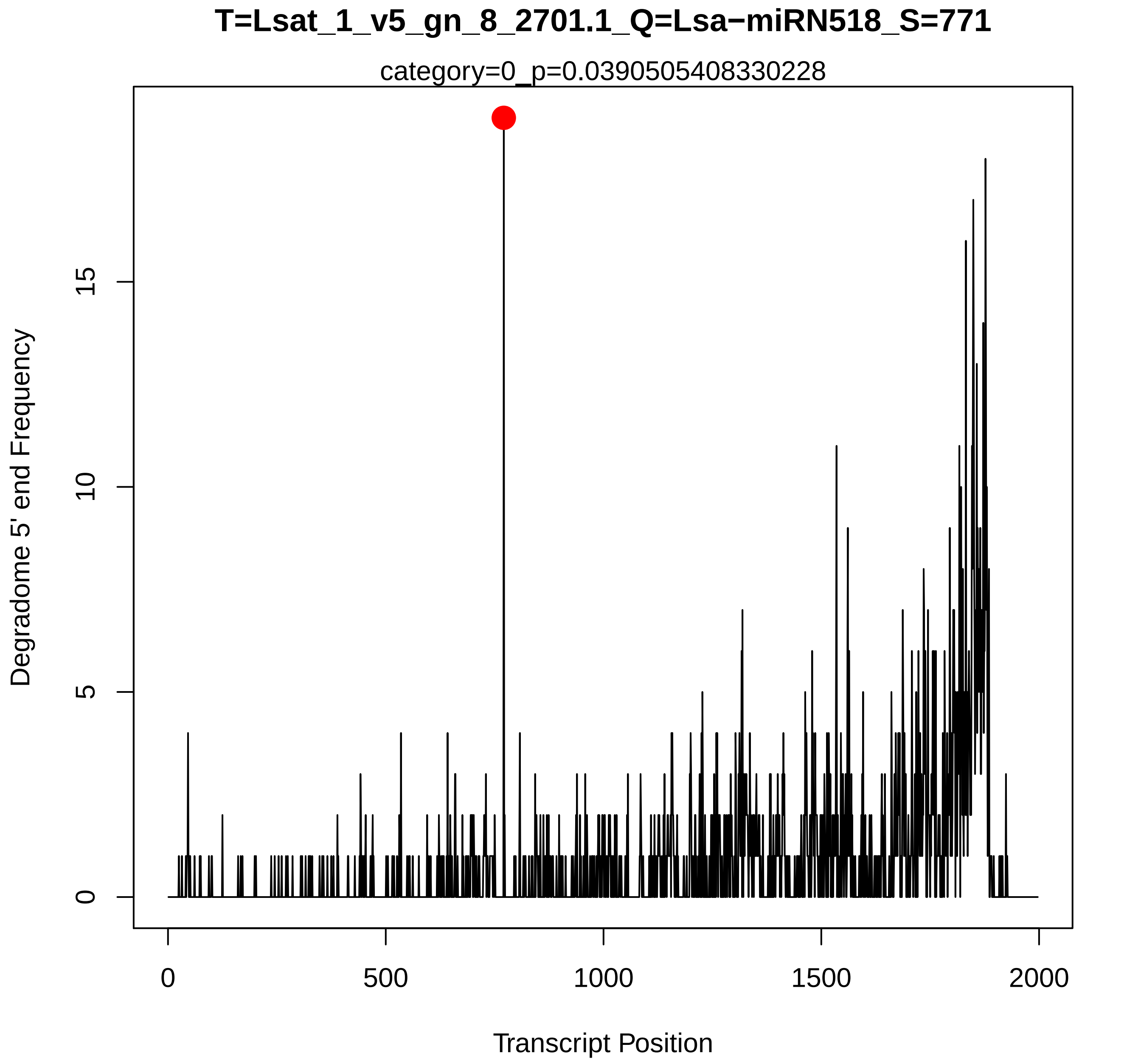

T=Lsat_1_v5_gn_8_2701.1_Q=Lsa−miRN518_S=771
category=0_p=0.0390505408330228
Degradome 5' end Frequency
0
5
10
15
0
500
1000
1500
2000
Transcript Position
